# Cardiac vagal activity and daily clinical practice

**Published:** 2016-02-23

**Authors:** Luc Quintin, John M. Karemaker, Robin M. McAllen

**Affiliations:** 1 Physiology, University of Lyon, Lyon, France; 2 Academic Medical Center at the University of Amsterdam, the Netherlands; 3 Florey Institute of Neuroscience and Mental Health, University of Melbourne, Australia

During most of the day heart rate (HR) is dominated by cardiac vagal (parasympathetic) activity – a fact taught in physiology classes and duly repeated at the appropriate exams. Only later in medical practice does one become aware of the power of cardiac vagal innervation, mainly in extreme cases: when it leads to syncope by too much or to tachycardia by too little activity, as in severe diabetes. In the previous issue, Karemaker [1] presents a coherent series of experiments to elucidate how the vagus nerve might control HR. The most important inputs for this control are bursts of baroreceptor afferent activity that are driven by the systolic rise in arterial pressure; emphasis was therefore put on burst-like vagal stimulation, as the expected outgoing reflex response of the vagus to the heart. The physician may ask, what is its significance for day-to-day medicine?

The anesthesiologist expects disappearance of fast beat-to-beat HR changes (HR variability) under inadequate analgesia [2]. This variability probably stems from the nucleus ambiguus of the medulla oblongata, the second relay station for the baroreceptor afferent traffic, where its activity may be modified by higher nervous centers (e.g. by a state of anxiety), by respiratory activity [3] and by input from the periphery [4]. Feeling the pulse in a young person may demonstrate clear-cut expiratory bradycardia, as hallmark of autonomic nervous health and proper functioning of the cardiac vagus (and, thereby, of the overall baroreflex). Striking in all vagally mediated HR responses is the unpredictability of the amount of the effect: the effect is known and expected to be there, but how much exactly HR will change seems to be a matter of chance. Karemaker explains part of this riddle by studying the relation between the timing of vagal bursts and the resulting HR responses. Even in this preparation, where both vagi have been cut and only the peripheral ends are stimulated, the effect on the rhythm of the heart is not precisely predictable. The culmination of this unpredictability was found when vagal bursts were delivered in each heart beat (like the predicted output from the baroreflex) at a critically late timing in the sinus node cycle, where the effect just might or might not reset the ongoing depolarization of the sinus node (Figure 1 in [1]). Then an ‘alternans’ in HR was observed, alternating short and long R-R intervals, where the small respiratory rhythm disturbance due to the external ventilator was now exaggerated. The effects of adding or suppressing just a few stimuli in a burst were strongest in that same critical time window. This phenomenon contributes to the unpredictability of vagal effects in real life. Other possible sources of ‘noise’ in HR are mentioned (but not further explored), such as the inherent instability of the autonomic transmission in the cardiac ganglionic plexus and membrane noise of the pacemaking process in the sinus node.

The rapid response to changes in composition of the vagal burst, even within the same heartbeat when heart rate is low, optimizes the effectiveness of the baroreflex to buffer blood pressure changes. Figure 1 in [1] explains this by showing how an increased systolic pressure leads to lengthening of the ongoing R-R interval, and consequently to stabilization of the next diastolic value. This is a key observation, basic to the ‘DeBoer’ model of fast baroreflex control [5]. This effect is diminished at higher HR, when a vagal burst only has its effect on subsequent heartbeats; but it is still present even during strenuous exercise [6]. However, with increasing age, hypertension, cardiac pathology, this rapid parasympathetic buffering gets lost. Eventually one finds a ‘fixed heart rate’ where sympathetically mediated changes are the only ones left. In elderly patients or patients with congestive heart failure, this has implications in the setting of hypotension and/or syncope: in the baseline state with minimal cardiac parasympathetic activity, acute hypotension can cause no cardiac parasympathetic withdrawal. Also, cardiac sympathetic activity is already high and has little capacity to further increase HR and cardiac output to buffer the hypotension. This explains part of the incidence in hypotension in elderly patients. Can the physician do something about this and, possibly, restore the parasympathetic buffering mechanism?

Pharmacologically pirenzepine, a muscarinic M1-antagonist, is known to increase time-domain indices of heart rate variability (pNN50, etc.) and baroreflex sensitivity (BRS) in patients after recent myocardial infarction [7]. Could manipulation of cardiac vagal activity be of help in preventing ventricular arrhythmias, especially in the sub-population with depressed BRS after myocardial infarction [8], synergistically with beta-blockade? Interestingly, alpha-2 agonists increase the phasic discharge of cardiac vagal motoneurons (i.e. the number of action potentials within one burst, as reproduced by Karemaker in his experiments) [9], lowering pressure lability [10]. This action is due to enhancement of baroreceptor afferent traffic [9] and probably also to central facilitation of the reflex, perhaps at the cardiac vagal motoneurons themselves. Accordingly, an alpha-2 agonist, clonidine increases HR variability and lowers systolic pressure variability, in hypertensive patients after major surgery (Figure 3 in [11]): it combines vasomotor and cardiac sympathetic de-activation with cardiac parasympathetic activation. The cardiac vagal motoneurons discharge more strongly, delivering more action potentials in each baroreceptor-triggered burst [9]. The mean R-R interval is longer, which means that the vagal efferent burst triggered by the previous systole arrives at the sinus node at an earlier phase of the cardiac cycle, at time when it can delay the next depolarization [1] and thereby buffer blood pressure fluctuations within a single R-R interval [5,10,12]. More generally, manipulation of the cardiac parasympathetic system, e.g. via M3 agonists, is a neglected issue in the setting of hypertension research [13].

In the field of medical device-based approaches (“electroceuticals”), stimulation of the vagus nerve is used to lower HR if nothing else works in the setting of chronic heart failure [14]. The Karemaker study points out how this can be done most effectively: in short bursts, coupled to the QRS-complex, early in diastole, beat-by-beat. This phasic stimulation is superior to the presently used continuous stimulation frequency, which gave very variable effects in the rabbit preparation [1].

The cardiac effects of the vagus nerve are considered a mirror of the soul, for instance when it gives away mental secrets that should have remained hidden in the lie detector test. The present study has lifted a piece of the veil by showing how the effector machinery (vagus nerve and sinus node) can produce fast, within one beat, changes in heart rate, and thereby stabilize blood pressure.
